# Malaria Prophylaxis: A Comprehensive Review

**DOI:** 10.3390/ph3103212

**Published:** 2010-10-13

**Authors:** Francesco Castelli, Silvia Odolini, Beatrice Autino, Emanuele Foca, Rosario Russo

**Affiliations:** 1 Institute for Infectious and Tropical Diseases, University of Brescia, Piazza Spedali Civili, 1 - 25123–Brescia, Italy; 2 Institute for Infectious Diseases, University of Catania, Via Palermo 635 – 95100 Catania, Italy

**Keywords:** malaria, prophylaxis, traveller

## Abstract

The flow of international travellers to and from malaria-endemic areas, especially Africa, has increased in recent years. Apart from the very high morbidity and mortality burden imposed on malaria-endemic areas, imported malaria is the main cause of fever possibly causing severe disease and death in travellers coming from tropical and subtropical areas, particularly Sub-Saharan Africa. The importance of behavioural preventive measures (bed nets, repellents, *etc.*), adequate chemoprophylaxis and, in selected circumstances, stand-by emergency treatment may not be overemphasized. However, no prophylactic regimen may offer complete protection. Expert advice is needed to tailor prophylactic advice according to traveller (age, baseline clinical conditions, *etc.*) and travel (destination, season, *etc.*) characteristics in order to reduce malaria risk.

## 1. Introduction

Malaria, transmitted by female *Anopheles* mosquitoes biting during night time, from sunset to dawn, is the most important parasitic disease worldwide. Five species (*Plasmodium falciparum, Plasmodium vivax, Plasmodium ovale, Plasmodium malariae* and, as recently discovered, *Plasmodium knowlesi*) can cause disease in humans. Clinical features and geographical distribution are reported in Table 1 [1,2,3,4,5,6].

**Table 1:** Clinical features and geographical distribution of Plasmodia species (reference [6], modified).

Species	Clinical features	Endemic areas (decreasing prevalence)
*Plasmodium falciparum*	Tertian non-relapsing malignant malaria	Sub-Saharan Africa, Latin America, South-East Asia
*Plasmodium vivax*	Tertian relapsing benign malaria	South-East Asia, Latin America, Sub-Saharan Africa
*Plasmodium ovale*	Tertian relapsing benign malaria	South-East Asia, Pacific, East Africa
*Plasmodium malariae*	Quartan non-relapsing benign malaria	West Africa, Guyana, India
*Plasmodium knowlesi*	Quotidian. Severe malaria may occur	South-East Asia

At first, malaria symptoms may be unspecific, including joint pain, asthenia and abdominal pain; followed by high fever, shivering, anorexia and vomiting. The most severe form is caused by *Plasmodium falciparum*, in particular in children, pregnant women and non-immune travellers from non-endemic countries.

People living in endemic areas progressively acquire a semi-immune status after repeated infections, reducing acute infection symptoms and disease severity. Nevertheless, after several years spent in non-endemic countries, immunity may be partially lost, as occurs in migrants from endemic countries to Europe or North America [7].

In a given area, malaria is defined *stable* when infection’s prevalence is constantly high during the whole year, conferring immunity to resident people; *unstable* when infection prevalence changes over years. According to parasitological and clinical indexes, areas may be classified in hypo-endemic, meso-endemic, hyper-endemic and holo-endemic (Table 2) [8].

**Table 2:** Classification of endemic malaria (reference [8]).

Type	Spleen rate	Parasite rate	Description
Hypo-endemic	Not exceeding 10% in children aged 2-9 years	Not exceeding 10% in children aged 2-9 years but may be higher for part of the year	Areas where there is little transmission and the effects, during an average year, upon the general population are unimportant
Meso-endemic	Between 11% and 50% in children aged 2-9 years	Between 11% and 50% in children aged 2-9 years	Typically found among rural communities in subtropical zones where wide geographical variations in transmission risk exist
Hyper-endemic	Constantly over 50% in children ages 2-9 years; also high in adults (over 25%)	Constantly over 50% among children aged 2-9 years	Areas where transmission is intense but seasonal and where the immunity is insufficient in all age groups
Holo-endemic	Constantly over 75% in children ages 2-9 years, but low in adults	Constantly over 75% among infants aged 0-11 months	Perennial, intense transmission resulting in considerable degree of immunity after early childhood

According to the World Health Organization (WHO), more than 100 tropical and sub-tropical countries in four macro-areas (Sub Saharan Africa, Latin America, Middle and Far East, Indian Subcontinent) are endemic for malaria and as many as 3.3 billion people (50% of the world population) are exposed to malarial infections. As many as 243 million malarial cases were estimated during 2008, mostly in Africa (85%), South East Asiatic countries (10%) and the East Mediterranean region (4%). Estimated malarial deaths during 2008 were 863,000 and 89% of them occurred in Africa, 6% in the East Mediterranean region and 5% in South East Asiatic countries [9].

According to the World Tourism Organization (WTO), an increasing number of international travellers is being reported, from 50 million/year after second World War to 922 million/year during 2008 [10,11] Approximately 80 million persons from industrialized nations travel to the tropical world each year [12]. Imported malaria mostly occurs in tourists and migrants travelling to their origin countries to visit friends and relatives [13].

In the last ten years, a decreasing trend in the number of malaria cases has been recorded in some African and South East Asian countries [14]. In India, a reduction from 260 cases/100,000 inhabitants in 1992 to 180 cases /100,000 inhabitants in 2005 was reported [15]. A similar decreasing trend, in the number of imported malaria cases, has recently been observed in the number of imported malaria cases in Europe [16]. In spite of that, malaria remains the main cause of fever in travellers from industrialized countries to endemic areas. Every year about 30,000 international travellers develop malaria, resulting in approximately 150 deaths [17].

Risk in non-immune travellers may be assessed by a mathematical model (a variant of the Mc-Donald model) on the basis of destination, season and malarial epidemic cycle. For example, travelling to the Amazon between December and February for a period of 120 days is ten times more risky in comparison of travelling to the same place and for the same period between June and August [18].

To assess malarial risk, the following variables are to be taken into account: destination, season, style and duration of the trip, *Plasmodium* drug sensitivity in the area, traveller’s baseline health conditions and local medical assistance [19]. Some studies underlined the strong association between trip’s duration and health risks [20], therefore travellers are usually divided into two categories: *short term travellers* and *long term travellers*. However, the time limits of the definition of *short-term travellers* are unclear, ranging from three weeks [21], less then one month [20], some weeks or few months [22] to less then six months [6]. *Short term travellers* are mostly tourists and businessmen coming from industrialized countries and represent 80-90% of travellers in tropical countries. *Long term travellers* commonly stay longer then six months [6,20,22] and they are mostly represented by male workers, missioners or researchers. Although they frequently received adequate counselling before leaving, their compliance to prophylactic measures tends to decline over time [20].

Travellers at high risk for imported malaria are represented by immigrants in industrialized countries going back to their origin countries to visit friends and relates (VFR). A high proportion of imported malaria cases occurs in this category [23,24]. According to the Geosentinel surveillance system, they have a higher risk of malaria and other vector-borne diseases in comparison with others travellers [25]. An American study showed that 90% of malaria’s diagnosis in the USA during 1997 was in people born in endemic areas [26]. This data are confirmed by a later study from the USA, which assessed that 64.5% of patients with malaria’s diagnosis were recorded in travellers born in endemic area visiting friends and relatives [14]. A European study on imported infectious diseases in Europe demonstrated that malaria is particularly frequent in migrants in Italy and France. According to the same study, migrants don’t receive adequate information before departure and have lower compliance to chemoprophylactic measures in comparison with European travellers [27], as also suggested by other studies [14,28,29]. Scolari and co-workers reported that 80% of migrants do not have adequate information and do not take preventive measures during travel, although they are aware of the malaria risk in their origin countries [30]. Sometimes they believe to be immune to malaria attack, thus decreasing their willingness to comply with preventive measures [23,31]. Therefore, raising awareness of the malaria risk in origin countries in migrants’ communities must be considered a priority in order to promote safer preventive behaviour when travelling. This is particularly true when VFRs are accompanied by children, who are at higher risk of severe malaria [14,24,30].

According to WHO guidelines, all travellers to high endemic malarial areas should be adequately informed on malaria risk, mosquito bite protection measures, chemoprophylaxis and stand by emergency treatment in case of evocative symptoms.

## 2. Anti-Vectorial Measures

Malaria is transmitted by the infected female *Anopheles* mosquito that inoculates *Plasmodium* sporozoites during blood feeding. They only feed during night (from dusk to dawn) when mosquitoes bite prevention is imperative [6]. All travellers to high malarial risk areas should be adequately educated on personal preventive measures, including behavioural prevention (long sleeved clothes, covering trousers and shoes, sleeping in air conditioned rooms), chemical prevention (repellents and insecticides), mechanic prevention (bed nets, window screens) [21].

### 2.1. Behavioural prophylaxis

Night hours are the target hours for protection against mosquito bites. Covering clothes (long sleeves clothes and covering trousers, preferably white) or light colours are a barrier for mosquito bites [6]. Insect repellents applied on clothing are effective for longer than they are on skin and extra protection is provided by treating clothing with permethrin or etofenprox [10]. Feet should be protected by appropriate footwear and by tucking long trousers into the socks [10]. It is also important to screen windows, to use air conditioners and to avoid staying close to freshwater pools and lakes [6,10].

### 2.2. Repellents and insecticides

Arthropod-borne diseases, including malaria, are the main causes of morbidity and mortality worldwide and more than 700 millions people are affected every year [32]. Repellents, causing alterations to arthropod’s sensorial organs, prevent their bites. They are highly effective but their efficacy is limited in time, requiring periodical re-application (every 3 to 4 hours according to manufacturers’ instructions) to maintain efficacy.

There are two categories of repellents: synthetic products and essential, plant-derived oils. Repellents containing DEET (*N,N*-diethyl-3-methylbenzamide), IR3535 (3-[*N*-acetyl-*N*-butyl]-aminopropionic acid ethyl ester) or icaridin (1-piperidinecarboxylic acid, 2-(2-hydroxyethyl)-1-methylpropylester) are commonly used [10]. DEET has been on the market since 1957; it has a broad spectrum of activity and many studies confirm a higher efficacy in comparison with both picaridin and *para*-methanediol (PMD). High concentrations of DEET (30-50% at least) guarantee a lasting protective efficacy. Uzzan and co-workers performed a randomized, double blinded, controlled study involving 100 volunteers of two villages in Senegal. They compared efficacy of four skin repellents (icaridine 20%, *para*-methanediol 20% and 50%, DEET 50%) with placebo, showing similar efficacy and duration of repellents’ action, statistically superior when compared with placebo. No differences were highlighted between men and women, between different species of *Anopheles* and no important adverse events were reported [32].

Repellents should always be considered for infants from two months of age going to highly endemic malaria areas [36]. WHO and CDC Guidelines approve their use, provided that recommended doses and concentrations are not exceeded. According to a recent and exhaustive review of the effectiveness of repellents to avoid arthropods’ bites, DEET is to be considered the gold standard while icaridin and PMD are adequate second choice alternatives [37]

### 2.3. Mechanic barrier

Impregnated bed nets remain the main tool against mosquito bites. A study performed in Uganda showed that sleeping under a bed net implies a lower risk of malarial infection, mostly in children, underlying the need to increase their use, particularly in rural areas of West Africa [38]. Many studies underline the importance of “*house screening*” control, including of windows, doors and gutters that could decrease indoor entry of mosquitoes [39]. The number of *Anopheles* caught in screened houseswas 59% lower in comparison with non-screened houses [40].

## 3. Chemoprophylaxis

Drug chemoprophylaxis has proved to be an effective preventive strategy in travellers to malaria- endemic areas [41], both for *P. falciparum* and *non-falciparum* malaria, despite it does not usually prevent the later relapses that can occur with *P. vivax* and *P. ovale*. Drugs can act on different stages of the *Plasmodium* biological cycle, on the pre-erythrocytic liver forms (*causal* prophylaxis) and on the erythrocytic blood forms (*suppressive* prophylaxis) [6]. Suppressive prophylaxis is mainly indicated for prevalent *P. falciparum* malaria areas, such as sub-saharian Africa, while causal prophylaxis is the best choice for *P. vivax* or *P. vivax* /*P.falciparum* malaria areas [42]. It is important to emphasize that no chemoprophylactic regimen confers complete protection and it must always be associated with primary prophylaxis aimed at avoiding mosquito bites. When drugs with exclusive suppressive activity are used, chemoprophylaxis must be continued for four weeks after the last possible exposure to account for pre-erythrocytic phase of the plasmodial cycle. Drugs with causal activity (atovaquone-proguanil or primaquine in primary prophylaxis) are to be continued for a shorter time (seven days).

### 3.1. Drugs for malaria chemoprophylaxis

#### 3.1.1. Chloroquine

It is a 4-aminoquinoline that interferes with parasite haem detoxification [1]. It has been the corner-stone of anti-malarial drugs up to the 80s, thanks to its efficacy, tolerability and safety in pregnancy and childhood. It is a rapid blood schizonticide recommended, in combination with proguanil, in areas dominated by *P. vivax* or in areas where *P. falciparum* is still sensitive to chloroquine, such as Central America and Middle Eastern countries [6].

It is effective against *P. malariae* and against erythrocytic forms of *P. vivax* and *P. ovale,* although reports on the appearance of genetic mutations conferring resistance to chloroquine have recently appeared [1]. When administered orally, blood peak concentration is reached in 1-6 hours. Its half-life, initially of 3-6 days, becomes 12-14 days with protracted intake. It’s metabolised in di-ethyl-chloroquine and it is excreted largely unchanged by the kidney. Chloroquine chemoprophylactic regimen in adults is 300 mg base/week (5 mg base/Kg/die in children) starting one week before the trip up to four weeks after return [43] (Table 3). It can induce several side effects, usually mild [44], including insomnia, nausea, headache, dizziness, blurred vision and itching. A cumulative dose of 100g may result in retinal toxicity, generally after 5-6 years of weekly intake [45]. Rare cases of central nervous system toxicity with seizures and mental disorders, myopathy, hearing loss, photosensitivity, hair loss and bone marrow suppression with aplastic anemia have also been reported [1]. Some studies have demonstrated its potential role in inducing psychosis [46] or “*non convulsive status epilepticus*” [47]. These findings emphasize that chloroquine should be used with caution in patients with a history of epilepsy.

In a study carried out on 6216 Peace Corps Volunteers in malaria endemic countries undergoing antimalarial prophylaxis for more than six months with chloroquine *versus* mefloquine *versus* doxycycline, chloroquine presented the safest profile, with lower incidence of serious adverse events requiring prophylaxis modifications in comparison with mefloquine and doxycycline [48]. Interactions with other drugs are very unusual. There is an increased risk of arrhythmias if chloroquine is administred concomitantly with drugs that prolong QT interval such as halofantrine. An increased risk of seizure or acute dystonia has been reported in association with mefloquine and metronidazole, respectively. Chloroquine reduces the bioavailability of both ampicillin and praziquantel and the therapeutic effect of thyroxine. On the contrary, it increases plasma concentrations of cyclosporine. It has a possible antagonist effect if administered together with carbamazepine and sodium valproate [1].

The first case of *P. falciparum* resistance to chloroquine has occurred in Thailand and Cambodia in the late 50s, only 12 years after the introduction of the drug. Since 1980, *P. falciparum* strains resistant to chloroquine have spread in South America, and since 1989 in Asia and Oceania. In Africa, chloroquine resistance of *P. falciparum* emerged in 1978 in the eastern areas and it has then spread to the whole of the African continent in the 80s. Currently, resistance to chloroquine is widespread in *P.falciparum* endemic areas, with the exception of Central America and the Caribbean. The parasites with greater resistance are generally those harbouring Lys76Thr and Ala220Ser mutations in PfCRT and Asn86tyr mutations in PfMDR1 [1]. Pfmdr1 duplication suggests a possible cross-resistance with mefloquine, which needs further studies to be confirmed [49].

#### 3.1.2. Proguanil

It’s a primary tissue schizonticide acting on the hepatic pre-erythrocytic cycle by inhibiting the parasite dihydrofolate reductase. It is only recommended in combination with chloroquine or atovaquone. It can’t be used alone in any malarious areas for the rapid onset of drug resistance.

When it is administered orally, its bioavailability is 60% and the blood peak is reached in 1-6 hours. It has a short half-life (13-15 hours), requiring daily administration. It’s metabolised in the liver to the active antifolate metabolite cycloguanil. Rarely, it can induce mild side effects such as anorexia, nausea and diarrhoea. It can cause oral ulcerations.

#### 3.1.3. Chloroquine-Proguanil

It’s a recommended prophylactic combination for travellers to *P. vivax* endemic areas with low *P. falciparum* prevalence with no widespread chloroquine resistance. In a prospective multicentre randomized trial among Dutch travellers to east, southern and central Africa no difference in prophylaxis failures between chloroquine 300 mg weekly and proguanil 100 mg daily, chloroquine 300 mg weekly and proguanil 200 mg daily and proguanil alone 200 mg daily regimens was found [50]. However the study was published in 1993 and the situation has changed since. The combination chloroquine-proguanil is no longer widely used but it is still occasionally considered for travellers visiting West Africa and for pregnant women [51]. This is also confirmed by a Danish study which showed that malaria cases in those who took chloroquine/proguanil were not due to poor compliance, as it was the case for those taking mefloquine, but were more probably due to the presence of drug resistance [52]. This drug combination can induce gastrointestinal side effects, epigastric pain, vomiting, oral ulcerations and blurred vision in long-term travellers above all [44]. In a 2003 multicentre, randomised, double blind, four arm study on a total of 623 non-immune individuals, the highest incidence of side effects and the highest incidence of moderate to severe skin problems and withdrawal was reported in the combined chloroquine-proguanil arm in comparison with mefloquine, atovaquone-proguanil and doxycyxline arms [53]. Exacerbation of psoriasis has been reported after about 2-4 weeks from initiation of chemoprophylaxis with chloroquine/proguanil [54] or chloroquine alone [55]. Nevertheless, given the severity of malaria and the low risk of reactivation of psoriasis during prophylaxis, the use of chloroquine/proguanil in patients with psoriasis is not strictly contraindicated if alternative regimen are not available, provided that the traveller is duly informed about the risk [56].

#### 3.1.4. Atovaquone-Proguanil

It is a combination of erythrocytic (atovaquone) and tissue schizonticide (proguanil) drugs. Atovaquone kills malaria parasites by inhibiting pyrimidine synthesis and plasmodial mitochondria electron transport at the level of the cytocrome bc1 complex [57], while proguanil acts on the hepatic pre-erythrocytic cycle by inhibiting the parasite dihydrofolate reductase; it has synergistic effect with atovaquone [58,59]. 

Adult chemoprophylactic regimen consists of 250/100 mg (1 adult tablet) atovaquone/proguanil/day (doses in children vary depending on weight) starting 1-2 days before the trip until 7 days after return [43] (Table 3). The combination offers a good balance of efficacy and tolerability against *P. falciparum* in non-immune travellers [6,60] and it is recommended in *P. falciparum* chloroquine-resistance areas [60]. Four randomized placebo-controlled trials reported a 97-100% efficacy of a fixed combination of atovaquone (250 mg) and proguanil (100 mg) in preventing *P. falciparum* malaria in semi-immune long-term residents in high malaria endemic areas of Africa. Atovaquone-proguanil has also demonstrated causal prophylactic action on the tissue forms of *P.falciparum* [59] while the protective efficacy against *P. vivax* has been shown to be moderate (84%) [58,59]. In a randomised study on healthy volunteers under aircraft cabin pressure conditions, atovaqone/proguanil has caused mild side effects such as nausea, vomiting, abdominal pain and stomatitis, but it did not cause insomnia or impaired ability to process information or impaired degree of attention and vigilance [58,61]. Indeed, atovaquone/proguanil showed a good comparative safety profile [22], despite the sporadic report of severe acute hepatotoxicity [62] and of a single case of Stevens-Johnson syndrome in a 65 year old traveller without other risk factors [63].

Due to its cost, VFRs travellers are sometimes reluctant to use this combination [60,64]. It’s registered in some countries for use not exceeding 28 days, but several studies have shown its safety and tolerability in travellers who have taken the drug for longer periods [65,66]. Data supporting its safety in pregnancy are insufficient [60] and the drug is contraindicated in children weighting less than 11 kg (WHO recommendation), lactating women, patients with varying degrees of renal impairment and patients with previous allergic reactions to atovaquone or proguanil [41].

If used alone, both atovaquone and proguanil can easily induce resistance in plasmodia, but the risk is significantly reduced when the drugs are given in combination [6]. Cases of therapeutic failure of atovaquone-proguanil have been reported, mainly in Africa [67], due to a point resistance mutation in the atovaquone mithocondrial cytb gene target of *P. falciparum* on codon 268 (ie Tyr268Ser, Tyr268Asn and Tyr268Cys). In a study conducted in three endemic areas in Thailand, *P. falciparum* isolates containing the mutation were not detected, thus supporting the use of the drug for both treatment and prophylaxis as the first choice in multidrug resistant areas in Thailand [57].

Chemoprophylactic failures of atovaquone/proguanil against *P. vivax* and *P. ovale* have been reported in travellers to Southest Asia, probably due to drug resistance [59]. Atovaquone/proguanil has proved effective in preventing *P. falciparum* imported malaria in English travellers although comparative data among the different recommended prophylactic regimens are limited [68].

#### 3.1.5. Mefloquine

It is a methanol-quinoline drug. As a blood schizonticide, it is very effective against *P. falciparum* and others *plasmodial* infections in malarial endemic areas, with the exception of some focal areas of South East Asia and Africa [21,41]. Its mechanism of action is not completely understood, probably similar to that of quinine but with a slower action. After oral administration, blood peak concentration is reached after 2-12 hours. Mean half life of mefloquine is 14-27 days, therefore it can be administered weekly. It is metabolised in the liver and less than 10% is excreted unmodified in urine. Mefloquine chemoprophylactic regimen in adults is 250 mg weekly, starting 1 or 2 weeks before departure and ending 4 weeks after return from endemic areas [43] (Table 3).

Neuropsychiatric adverse events may occur during mefloquine chemoprophylaxis, from nightmares to psychosis, that might require prophylaxis interruption [41,69]. Usually, neuropsychiatric adverse effects occur after 2-3 doses, mostly in subjects with history of neuropsychiatric disturbances; such problems should carefully be ruled out before prescribing mefloquine [41]. Neuropsychiatric disturbances after mefloquine intake are more frequent in women [53] and in people under 34 years of age [70].

A randomised, double blind, controlled study compared all currently available prophylactic regimens in 623 travellers. Mild to moderate side effects were reported in 45% of chloroquine/proguanil recipients, in 42% of those taking mefloquine, in 33% of those taking doxycycline and finally in 32% of those under atovaquone/proguanil prophylactic regimen. Severe adverse events were reported by 11%, 12%, 6% and 7% in those travellers taking mefloquine, chloroquine/proguanil, doxycycline or atovaquone/proguanil respectively [53]. Among rarest adverse events, eosinophilic pneumonia in a woman [69] and one case of reversible tinnitus and hearing loss in a 67 years old women with history of broken tympanum in childhood [71] have been reported. Mefloquine adverse events may discourage travellers from the use of this effective drug, even if discordant evidence exists [48,72]. Conversely, a multicenter prospective study performed in 2007 in Switzerland showed that 50% of people choose mefloquine (in spite of the fact that they received written information also about atovaquone/proguanil and doxycycline) and two third of travellers that used mefloquine in the past, chose it again [73]. Mefloquine-associated disturbances are due to personal hypersensitivity and travellers who do not report side effects during their first mefloquine use will probably not do so even during subsequent use. The failure of mefloquine chemoprophylaxis principally depends on poor compliance rather than resistance [52].

Nevin suggests that mefloquine (prophylactic dosage) could accumulate in the brain tissue and alter calcium homeostasis and gap junctions. These effects may produce convulsions, myoclonus and ataxia in predisposed people, including those carrying the EPM1 mutation (*Progressive Myoclonic Epilepsy Type 1*). This study suggests avoiding mefloquine use in individuals who have experienced myoclonus and ataxia or in subjects with confirmed EPM 1 mutation. In these cases an alternative regimen (atovaquone/proguanil or doxycycline) should be used [74,75]. Travellers may have a better adherence to mefloquine prophylactic regimen in comparison with chloroquine/proguanil, probably due to its weekly administration [44]. Conversely, a better adherence to atovaquone/proguanil regimen in comparison to mefloquine has also been reported by other studies, probably because of its shorter duration of administration after return [66,76].

When mefloquine is well tolerated after the first weeks of administration, it is generally well tolerated also for a longer period. In an historical prospective study performed on 5120 Italian soldiers deployed in Somalia and Mozambique in 1992-94, mefloquine was well tolerated for as long as six months [77]; the same was observed in Peace Corps Volunteers up to 2.5 years [78]. Significant side effects were reported in only 0.3% German sailors who took mefloquine for six months [45].

#### 3.1.6. Doxycycline

It is a semi synthetic derivative belonging to the tetracycline family, with a good pharmacokinetic profile. It is well absorbed orally with a good penetration into tissues and body fluids. It acts on ribosomes (30S subunit) by blocking protein synthesis. It is active against many bacteria and it has also an anti-inflammatory activity. It has a short half-life (14-35 hours), requiring daily administration starting from 1-2 days before departure until four weeks after return from endemic areas [6,43] (Table 3). It is used in combination with quinine for the treatment of malaria and alone for chemoprophylaxis in *P. falciparum* chloroquine-resistant areas even if cases of prophylactic failures are reported [79]. It is the drug of choice, together with atovaquone/proguanil, in Southeast Asia areas where multiple drugs resistance strains are reported (northern Thailand, Myanmar border areas, Cambodia and Laos), when mefloquine is contraindicated or not tolerated. Usually, doxicycline is well tolerated. However, it may cause epigastric pain, nausea, diarrhoea, abnormal vaginal flora and intestinal esophagitis [41]. Photo-toxicity is dose-dependent and requires careful sun protection [41]. In a study performed on 35 patients with Q fever endocarditis, twenty-one patients were treated with a combination of doxycycline (100 mg twice daily) and hydroxychloroquine for an average of 31 months, and all these patients experienced photo-toxicity, mainly on hands and nose, but only one patient had irreversible skin pigmentation caused by doxycycline treatment [80]. Among 153 travellers under doxycycline chemoprophylaxis, one third had skin reactions and no episode of vaginal candidiasis was reported [53]. It is contraindicated in pregnant or breastfeeding women and in children under 8 years of age [81]. Doxycycline absorption is reduced by anti-acids and iron while its metabolism is accelerated by liver enzymes inducers drugs (rifampicin, carbamazepine, phenytoin, phenobarbital) or by alcohol abuse [1].

#### 3.1.7. Primaquine

It interferes with *plasmodial* mitochondrial system by acting on electron transport. When administered orally, the blood peak concentration is reached in 2-3 hours. It has a short half-life of about seven hours. It’s metabolised in the liver and only a small percentage of the drug (< 1%) is eliminated in the urine. Primaquine is widely distributed into body tissues [1]. Its therapeutic use for the treatment of *P. vivax* and *P. ovale* infections was approved by the Food and Drugs Administration (FDA) in 1952. Several studies have shown that it is well tolerated and effective as primary prophylaxis against *P. falciparum* and *P. vivax*, when administered at a dose of 30 mg daily in adults, from the day before departure until seven days after return [41,82,83]. It acts on the pre-erythrocytic cycle of the parasite. Even if the prophylactic use of primaquine is not officially approved by FDA changes in labelling to include its preventive use against *P. falciparum* and *P. vivax* malaria have been considered [84]. Its use as second line drug in chemoprophylaxis is proposed by the US CDC only in selected circumstance for travellers who are unable to take others first line drugs [43]. Primaquine is the only drug which acts on hypnozoites, and it is the drug of choice for *P. vivax* and *P*. *ovale* terminal prophylaxis (presumptive anti-relapse therapy overlapping with a blood schizonticide) in long term travellers at high risk for these infections [21,43]. Terminal prophylaxis in adults is done taking 30 mg of primaquine for 14 days starting the first day after return. A study showed that the first *P. vivax* attack may occur three months after returning from endemic areas, regardless of the use of chemoprophylaxis [22]. It is active against hepatic hypnozoites, though the increasing incidence of primaquine-resistant *P. vivax* strains in Oceania, South-East Asia and South America represents a challenge [85]. Clinical trials indicate that the efficacy of primaquine is higher than 95% as terminal prophylaxis at doses of 30 mg daily for 14 days (in combination with a blood schizonticide such as chloroquine) and greater than 85% as prophylaxis [86]. It can induce life threatening haemolitic anemia in patients with G6PDH deficiency. It may also cause gastrointestinal side effects such as abdominal pain and nausea that are mitigated by food. Psychomotor and neuropsychiatric disorders are not usually reported with the exception of one case of depression and psychosis [87]. Several authors emphasize the importance of informing travellers from areas endemic for *P.vivax* and *P.ovale* of the possible risk of disease even long after the end of chemoprophylaxis [22].

#### 3.1.8. Tafenoquine

It is an 8-aminoquinoline which is still under development. It has a long half-life of about 14 days. It is generally safe and well tolerated, except for pregnant women and for people with G6PDH deficiency. In several studies, tafenoquine was effective both as causal and suppressive prophylaxis for *P. vivax* and *P. falciparum*. Several studies carried out in Africa have shown its effectiveness in suppressing *P.falciparum* parasitaemia by weekly administration. A randomized, controlled, double-blind study made in Thailand on 205 soldiers demonstrated that monthly administration of 400 mg tafenoquine is effective in suppressing both *P.vivax* and *P. falciparum* parasitaemia. However, its causal prophylaxis activity was incomplete [88]. Tafenoquine may cause some mild to moderate side effects, mainly gastrointestinal (nausea and diarrhoea), moderate rise in transaminases (ALT> AST) and transient rise in creatinine values [88]. Overall, tafenoquine is a promising, effective and tolerated drug, representing a potential alternative to first-line drugs used in the prevention and treatment of malaria [41,87,88]. However in a recent randomized (3:1), double-blind, phase III Australian study, 93% of patients receiving 200 mg of tafenoquine weekly for a period of 6 months experienced the onset of keratopathy (vortex kheratopathy) without effects on sight and with complete resolution in all subjects within 1 year after drug withdrawal [89]. Screening for G6PDH deficiency is necessary in all patients taking tafenoquine in order to prevent drug-induced hemolysis. Women of childbearing age should undergo pregnancy tests and adopt measures to prevent pregnancy [88].

**Table 3:** Drugs used in the prophylaxis of malaria (refs. [1,43] modified).

Drug	Areas	Mode of intake	Adult dose		Pediatric dose	Pregnancy	Controindications
**Atovaquone-Proguanil^2^** (ATV/PGN)	All malarious areas	Start 1 day before entering malarious areas. Continue up to 7 days after leaving such areas	250/100 mg daily orally		Pediatric tablets (ped. tabs) containing 62.5 mg atovaquone and 25 mg proguanil hydrochloride: 11-20 kg: 1 ped. tab/die; 21-30 kg: 2 ped. tabs/die 31–40 kg: 3 ped. tabs/die 40 kg: 1 adult tab/die Not recommended under 11 kg because of limited data	Not recommended	Hypersensitivity, severe renal impairement (creatinine Cl < 30 mL/min), children < 11 kg
**Chloroquine** (CLQ)	*P. vivax. P. ovale, P. malariae* and CLQ-sentitive *P. falciparum* areas	Start 1 week before entering malarious areas. Continue up to 4 weeks after leaving such areas. If daily doses: start 1 day before departure	300 mg base/weekly (also when proguanil is associated)		5 mg/kg/weekly	Recommended	Hypersensitivity, epilepsy, psoriasis, retinal diseases, severe hepatic failure
**Doxycycline** (DOXY)	All malarious areas	Start 1 day before entering malarious areas. Continue up to 4 weeks after leaving such areas	100 mg/die		Controindicated under 8 years of age	Not recommended	Cutaneous hypersensitivity, hepatic diseases, hypersensitivity to tetacyclines
**Mefloquine^3^** (MFQ)	Prophylaxis in areas with mefloquine-sensitive malaria	Start 1 week before entering malarious areas (preferably 2-3 weeks). Continue up to 4 weeks after leaving such areas	250 mg base (1 tab)/ week		Not recommended under 5 kg because of lack of data.	Not recommended in the first trimester of pregnancy because of lack of data	Hyper-sensibility, seizures, psychiatric disorders, cardiac conduction abnormalities
**Primaquine^4^**	Prophylaxis for short-duration travel to areas with high risk of *P.vivax* or *P. ovale* malaria	Start 1-2 days before entering malarious areas. Continue up to 7 days after leaving such areas		30 mg base daily	0.5 mg/kg base daily up to adult dose	Not recommended	Gastrointestinal disorders, G6PDH^1^ deficiency
**Primaquine^4^** (terminal prophylaxis)	Use for terminal (anti-relapse) prophylaxis in areas at high risk of *P. vivax* or *P. ovale* malaria	Prophylaxis for 14 days after leaving malarious areas		30 mg base daily	0.5 mg/kg base daily up to adult dose	Not recommended	Gastrointestinal disorders, G6PDH^1^ deficiency

^1^ Glucose-6-phosphate dehydrogenase. All persons who take primaquine should have a documented normal G6PDH level before starting the medication; ^2^ CDC Guidelines: 5–8 kg: 1/2 ped. tab/die;                         9–10 kg: 3/4 ped. tab/die                         11–20 kg: 1 ped. tab/die                         21–30 kg: 2 ped. tabsl/die                         31–40 kg: 3 ped. tabs/die                         40 kg: 1 adult tab/die ^3^ CDC Guidelines: ≤9 kg: 4.6 mg base/kg weekly                         10–19 kg: 1/4 tab weekly                         20–30 kg: 1/2 tab weekly                         31–45 kg: 3/4 tab weekly                         ≥ 45 kg: 1 tab weekly ^4^ Not approved by FDA for prophylactic use

### 3.2. Recommendations according to geographical areas

The following recommendations by WHO Regions are provided. Because the epidemiology of malaria is extremely variable, this advice needs to be complemented by updated epidemiological information. 

#### 3.2.1. East Mediterranean region

There are six high transmission malaria countries (Afghanistan, Djibouti, Pakistan, Somalia, Sudan and Yemen) and three low and geographical limited transmission malaria (Iran, Iraq, Saudi Arabia) in this region. *P. falciparum* is the dominant species in Djibouti, Saudi Arabia, Sudan and Yemen, but *P. vivax* causes most of Afghanistan and Pakistan malarial cases and all of Iran and Iraq ones. As many as 890,000 malaria cases were registered in these regions during 2008. More than 90% of them were diagnosed in Afghanistan (7%), Pakistan (18%), Somalia (10%) e Sudan (62%). A clear decreasing trend was noted in four countries (Afghanistan, Iran, Iraq, Saudi Arabia), as a result of effective malaria control programmes [9]. The choice of prophylaxis should be guided by travel itinerary. 

#### 3.2.2. Sub-Saharan Africa

In most of Sub-Saharan Africa countries malarial transmission risk is high or very high, with a widespread chloroquine resistance. Mefloquine, atovaquone/proguanil and doxycycline are the first line drugs recommended for chemoprophylaxis in these areas [43]. Chloroquine/proguanil may also be considered in selected circumstances (early pregnancy, long term stays, *etc*), but its efficacy has dramatically decreased in many areas in the last decade. Season and altitude should be considered when advising chemoprophylactic drugs in travellers.

#### 3.2.3. South East Asia

Ten of eleven countries of this area are endemic for malaria and approximately 30% of the population is at high risk of infection (incidence: >1/1,000/year). As many as 2.4 million malaria cases and 2,408 deaths were reported in 2008 and 97% of them occurred in four countries (India 55%, Myanmar 17%, Indonesia 15%, Bangladesh 10%), mostly due to *P. falciparum.* There is a variability between predominant species in South East Asia regions: in Myanmar and Timor Leste the predominant specie is *P. falciparum*, while in the Democratic People’s Republic of Korea *P.vivax* infection dominates. In four countries (Bhutan, Sri-Lanka, Democratic People’s Republic of Korea and Thailand) a significant decrease of malarial cases has recently been observed, demonstrating the effectiveness of control programmes [9]. 

#### 3.2.4. West Pacific

Malaria epidemiology is very heterogeneous in this area. Papua New Guinea, Salomon and Vanuatu Islands are the countries with the highest transmission of chloroquine resistant *P. falciparum* and *P. vivax* worldwide. Malaria is also endemic in the Mekong regions, Cambodia, Yunnan (China), Laos Democratic Republic and Vietnam. Few cases occur in Malaysia and the Philippines. Most malaria cases are due either *P. falciparum* or *P. vivax*, while in China *P. vivax* is the only reported specie. As many as 240,000 confirmed cases were reported during 2008 and a progressive decrease of malarial incidence was reported in six countries (Laos Democratic Republic, Malaysia, Salomon and Vanuatu Islands and Vietnam) [9]. Doxycycline, atovaquone/proguanil and mefloquine may be used for chemoprophylaxis (except for Papua New Guinea: above 1,800 m, where risk does not exist), but *P. vivax* relapse’s risk is high.

#### 3.2.5. Central and South America

Malaria is endemic in 21 countries and 30% of people are exposed to malaria risk. As many as 77% of total reported cases during 2008 were attributed to *P. vivax* infection, but all cases in Haiti and Dominican Republic were caused by *P. falciparum*. Incidence of malaria in these countries considerably decreased, from 1.14 million in 2000 to 572,000 in 2008. Over 50% reduction was observed in nine countries (Argentina, Belize, El Salvador, Guatemala, Guyana, Mexico, Nicaragua, Paraguay and Suriname) [9].

#### 3.2.6. Europe

During 2008, six Countries belonging to the WHO European Region (Azerbaijan, Georgia, Kyrgyzstan, Tajikistan, Turkey e Uzbekistan) reported malaria cases. In Tajikistan only two cases of *P. falciparum* malaria were reported in 2008. In all other areas, *P.* *vivax* is the only authoctonous specie. Malaria transmission is seasonal and occurs between June and October with focal distribution. A dramatic decline in malaria cases was observed (except from Kyrgyzstan) from the year 2000 (32,474 cases) to the year 2008 (660 cases). Table 4 reports WHO chemoprophylactic recommendations by area.

**Table 4:** Prophylaxis by area (reference [90], modified).

Type	Malarial Infection risk	Prevention
I	Limited	Anti-vectorial measures only
II	*P.vivax* esclusively or chloroquine sensitive *P. falciparum*	Anti-vectorial measures and chloroquine chemoprophylaxis
III^1^	*P.vivax* and *P. falciparum*, with chloroquine resistance areas	Anti-vectorial measures and chemoprophylaxis with chloroquine/proguanil
IV	1) High risk of *P. falciparum* infection with reported drug resistance 2) Mild/moderate risk of *P.falciparum* infection with high levels of drug resistance^2^	Anti-vectorial measures and chemoprophylaxis with mefloquine, atovaquone/proguanil or doxycycline (on the ground of resistance pattern)

^1^ The areas where type III prevention is still an option are limited to Nepal, Sri Lanka and Tajikistan, and parts of Colombia and India. If necessary, Type IV prevention can be used instead. ^2^ Alternatively, in rural areas with high rate of drug resistance and low risk of *P. falciparum* infection, *stand-by emergency treatment* might be used instead.

## 4. Stand-By-Treatment

WHO defines *stand-by emergency treatment* (SBET) as the use of antimalarial drugs carried by travellers for self treatment when malaria evocative symptoms occur and medical care isn’t accessible within 24 hours from the onset of symptoms. WHO recommends SBET for selected categories of travellers, such us occupational traveller (es: aircraft crew: short but frequents stops in endemic areas), long-term travellers who opt for seasonal chemoprophylaxis and travellers in multi-drug resistant areas with low risk of malaria transmission [90].

Drugs used for SBET are those used for uncomplicated *P. falciparum* malaria: artemether-lumefantrine, atovaquone-proguanil, quinine plus doxycycline or quinine plus clindamycin. Individual dosages are reported in Table 5 [90]. A different drug from the one that was used in chemoprophylaxis, if any, should be considered for SBET [22]. SBET efficacy depends on it’s correct use and travellers may have difficulties appropriately adopt SBET; several studies [91,92] suggest that travellers may not have recourse to the treatment appropriately. Overtreatment is frequent: as many as 57% of those travellers who adopt SBET did not have malaria at all according to a recent paper [91]. Adequate counselling is needed to avoid SBET misuse. The use of self diagnostic rapid tests is controversial, because their quality may deteriorate in tropical countries where temperature and humidity are high [93]. Furthermore, travellers may experience problems with performance and interpretation of the test [91]. Counterfeit drugs is a crucial problem in developing Countries, particularly in Southeast Asia [94,95,96,97], suggesting that travellers should take drugs along from their origine country.

**Table 5:** WHO stand-by emergency treatment recommendations (ref. [90], modified).

Drug	Dosage
Artemether/Lumefantrine	6 doses in 3 days (taken at 0,8,24,36,48 and 60 hours)
	5-14 Kg: 20/120 mg per dose
	15-24 Kg: 40/240 mg per dose
	26-34 Kg: 60/360 mg per dose
	> 35 Kg: 80/480 mg per dose
Atovaquone/proguanil	1 dose/daily for 3 days
	5-8 Kg: 125/50 mg daily
	9-10 Kg: 187,5/75 mg daily
	11-20 Kg: 250/100 mg daily
	21-30 Kg: 500/200 mg daily
	31-40 Kg: 750/300 mg daily
	> 40 Kg: 1gr/400 mg daily
Clindamycin §	< 60 Kg: 5 mg base/Kg 4 times daily for 5 days
	> 60 Kg: 300 mg base 4 times daily for 5 days
Doxycycline §	adult > 50 Kg: 800 mg salt in 7 days (100 mg x 2 day 1; 100 mg days 2-7)
	children > 8 years:
	25-35 Kg: 50 mg per dose
	36-50 Kg: 75 mg per dose
Quinine	8 mg/base/kg 3 times daily for 7 days

§ To be used only in combination with quinine in areas of emerging quinine resistance.

## 5. Risk Groups

### 5.1. Long-term travellers

Long-term travellers, usually non-immune at departure, are defined as those travellers staying abroad for longer then six months [22,45,98]. Examples of long-term travellers are Peace Corps Volunteers, military personnel, students, teachers, researchers and missionaries. In comparison with short-term travellers, they usually have lower compliance to antimalarial prophylaxis for many reasons: complicated schedule, fear of side effects, lack of unambiguous information by medical doctor, low perception of real risk, unawareness of disease’s severity [22,47]. On the other hand, they have a higher exposure risk to malaria infection, proportional to the length of stay [91,99].

A combination of preventive measures is advisable (always including anti-vectorial protective measures) and highly effective. Unfortunately, studies on safety of long term use of repellents are lacking and these measures are poorly used [45].

Long term chemoprophylaxis with mefloquine is well tolerated and accepted because of few side effects and schedule simplicity [100,101] and its safety has long been documented on Peace Corps Volunteers in Sub-Saharan Africa [78]. Nevertheless, the risk of neuropsychiatric adverse events, particularly in women, is to be carefully assessed in each individual traveller [102,103]. A study by Nevin and colleagues found that 9.6% of USA military personnel deployed to Afghanistan had contraindications to mefloquine use [104].

In some countries, the use of atovaquone/proguanil prophylaxis is officially restricted to 28 days [96], although many studies confirm its safety and efficacy for longer period [105,106,107] and CDC Guidelines give no limits regarding the duration of its chemoprophylactic use. In comparison with mefloquine, atovaquone/proguanil presents two limits, which are cost and daily schedule. 

Another drug used in chemoprophylaxis is doxycycline, whose long-term safety is well demonstrate by studies concerning treatment of acne and Q fever. It is contraindicated in pregnant women and in children below eight years of age. 

In chloroquine sensitive areas, use of chloroquine/proguanil is a chance for long-term travellers. However, its prolonged use has been associated to maculopathy and retinopathy [108,109,110,111]. In case of prolonged use, regular ophthalmologic examinations are recommended.

Specific recommendations on long-term primaquine prophylactic use are lacking. Two studies suggest its safety and efficacy in prolonged use [112,113]. Limits of primaquine use are represented by daily schedule and its absolute contraindication in individuals with G6PDH deficiency. 

Adherence could be improved using alternative strategies such us stand-by treatment and seasonal chemoprophylaxis, both requiring good information and adherence. 

### 5.2. Children

Non-immune young travellers are at high risk of severe malaria when travelling to malaria endemic countries [23,24]. A recent study estimated the incidence of children’s imported malaria diagnosed in 18 hospitals of northern France (from 2000 to 2006) at 1.9/100,000 resident children under 18 years old [114]. VFR’s children are often born in malaria non-endemic areas and are exposed to a risk setting when visiting friends and relatives in their parents’ origin country, often without chemioprophylaxis or awareness of the disease

Often children’s prophylaxis is not regularly followed [23] and children may experience the most severe form of the disease with high-parasitemia [115]. It may not be overemphasized that prevention strategies for paediatric travellers should be strict, using both chemoprophylaxis and personal protection measures against mosquitoes [116].

When travel cannot be avoided or deferred, it is very important to use personnel protective measures, such us insect repellents and insecticide-treated nets and clothes [36,116,117]. WHO and CDC Guidelines recommend their use in children over two months of age, provided that the paediatric formulation is used. The French Group of Tropical Paediatrics recommends the following age-related schedule of topical repellent: once daily in children above two months; twice daily in children ages one to 12 years; three times daily after 12 years [36]. Topical repellents and insecticides are also useful to prevent other arthropod borne infections, such us Chikungunya, West Nile virus, Lyme borreliosis, dengue virus. Chemoprophylactic drugs may have age-related restrictions that have to be considered carefully by the prescribing physicians before travel (table 3). Breastfed children aren’t protected by the small amount of drug passed in milk and they should be administered their own regular chemoprophylaxis. Despite good compliance to preventive and chemoprophylactic measures, malaria diagnosis should always be promptly considered in children who experience fever after travel to endemic malaria area [114].

### 5.3. Pregnant and breastfeeding women

Malaria is a severe disease during pregnancy both for the mother and the foetus. It may cause hypoglycaemia, pulmonary oedema, cerebral malaria, haemolytic anaemia in non-immunes mothers [118,119], stillbirth and miscarriage in foetus, besides low birth weight and neonatal death [118,119,120]. Pregnant women should always be advised against travelling to malaria endemic areas, if possible. As well as children, pregnant women should use personal measures (repellents, insecticides) to prevent mosquito bites. DEET-based repellents are safe during pregnancy [122,123] and are recommended by WHO and CDC Guidelines during pregnancy, provided that recommended doses are not exceeded. Safety data of chemoprophylactic drugs during pregnancy are limited because pregnant women are often excluded from clinic trials for ethical reasons. Recommendations are therefore difficult to formulate in pregnancy, as drug’s pharmacokinetic and pharmacodynamics are modified. 

Primaquine and doxycycline are contraindicated [22,124]: primaquine causes intravascular haemolysis in G6PDH deficiency foetus and doxycycline is potentially teratogenic. 

There are limited data about safe use of atovaquone/proguanil in pregnancy. Both WHO and CDC Guidelines do not recommend its use. Chloroquine is safe during pregnancy, as confirmed by its long use and by a randomized, double-blind, placebo-controlled trial in pregnant Karen women that demonstrated that weekly chloroquine prophylaxis is safe for foetus and prevents *P. vivax* episodes [125]. Mefloquine is safe in the second half of pregnancy [126] and some authors recommend mefloquine use in high risk areas [127]. A post-marketing surveillance study evaluated the teratogenic potential of mefloquine in 1627 pregnant women, concluding that the risk wasn’t different from the risk observed in the general population [128]. Proguanil is safe during pregnancy, and folic acid 5 mg daily should be associated.

Quinine and chloroquine are considered safe SBET-choices by WHO. Mefloquine, artemisin and derivates may be used from the second trimester on, while artemether/lumefantrine and atovaquone/proguanil are not recommended because of lack of data [90].

Breastfeeding women may safely use mefloquine, chloroquine, hydroxychloroquine and proguanil [1]. As only a small amount of these drugs pass into human milk, they are considered safe for children [129]. Atovaquone/proguanil could be used in breastfeeding mothers of newborns over 5 kgs according to CDC, though WHO advices against its use because data are limited. Primaquine could be used only if the absence of G6PDH deficiency is documented, both in the mother and the child. 

### 5.4. Immunocompromised traveller

Thanks to new therapeutic strategies, the number of persons living with immune-compromising conditions is increasing as well as the number of immune-compromised individuals travelling to tropical countries. A recent study underlines the importance of anti-malarial chemoprophylaxis in all immunocompromised travellers for any reason, with particular regard to splenectomy and HIV infection [130].

Splenectomized and hyposplenic patients have an increased risk of severe malaria, due to absence or decreased spleen ability to clear malaria parasites [130,131]. A French study reported three fatal cases of malaria infection in splenectomized patients, despite intravenous quinine treatment [132]. These individuals should intensify personal protection measures and adhere carefully to chemoprophylaxis even for prolonged periods [133].

Thanks to highly active antiretroviral therapy (HAART) the number of people living and travelling with HIV/AIDS has increased in recent years [134]; a fatal case of malaria in an HIV positive traveller has been reported [135]. The interactions between malaria and HIV are complex; Abu-Raddad has assessed the reciprocal influence of HIV and malaria by means of a mathematical model in Kenya [136]. A study performed in Uganda has failed to demonstrate a significant increase in malarial episodes in HIV-infected compared with HIV-uninfected children. However, HIV-infected children experienced more severe episodes, higher hospitalization rate and more blood transfusions than the control group [137]. People with lower CD4 counts are exposed to a higher risk of severe malaria. WHO recommends a viro-immunological assessement (CD4 count, HIV viral load) with clinical examination before travel [10]. HIV infected pregnant women are the most at risk. The possibility to defer the travel after deliver should be considered. HIV infected travelers should start adequate prophylaxis before their trip, also considering interactions between antiretroviral and antimalarial drugs [134,138,139]. Mefloquine reduces some protease inhibitors levels (ritonavir with coadministration; probably atazanavir, lopinavir and nelfinavir); its plasma levels could be reduced by efavirenz and nevirapine. Atovaquone/proguanil levels could be reduced by indinavir, as well as ritonavir and lopinavir, efavirenz and atazanavir/ritonavir [140]; it could reduce indinavir levels and could increase zidovudine levels causing hematological problems [130]. No data are available on doxycycline interactions [134]. All these considerations should be taken before starting chemoprophylaxis and dosage should be arranged to avoid under or overexposure to the different drugs.

Atovaquone/proguanil might be a suitable SBET option for HIV-infected subjects receiving NNRTI-based regimen, while mefloquine could be considered as SBET drug by subjects taking PI-based HAART [141]. Antimalarial artemisinin action is augmented by HIV protease inhibitors indinavir or nelfinavir, as recently demonstrated *in vitro* [142].

## 6. Take Home Messages

1) Malaria still represents an important scourge in endemic areas, causing a relevant burden of morbidity and mortality in particular in children under five years of age and pregnant women;
2) Due to increased human mobility, imported malaria has emerged as a public health issue in non-endemic areas of western countries, requiring educational activities for physicians in non endemic countries. Persons visiting friends and relatives (VFRs) are at particular risk;
3) Malaria preventive measures are key to avoid malaria infection and diseases. Key preventive measures are anti-vectorial measures to avoid mosquito bite and adequate chemoprophylaxis to avoid diseases. The combination of these two approaches gives the best protection;
4) Adequate chemoprophylaxis must be tailored on the individual traveler, taking into account travel itinerary (destination, altitude, malaria epidemiology) and features (season, style of travel) and individual characteristics (age, baseline conditions, *etc.*).
5) Persons at increased risk of infection and severe disease include children, pregnant women, immunocompromised persons and long-term travelers, who require special attention;
6) No antimalarial prophylactic regimen gives complete protection;
7) In particular situations (low endemicity, repeated travels, persons unable to take chemoprophyactic drugs), stand-by antimalarial emergency treatment (SBET) may be considered.

## 7. Priorities for Future Research

1) To monitor the emergence of pharmacological resistance among international travellers to prevent loss of effectiveness of available drugs;
2) To implement research on new convenient and safe drugs for long-term travellers;.
3) To improve data on antimalarial drugs safety in pregnancy, in particular during the first trimester of pregnancy.
4) To explore drug interactions between antimalarial agents and new antiretroviral drugs or classes (Integrase Inhibitors, CCR-5 Antagonist).
